# Multifunctional Textiles with Flame Retardant and Antibacterial Properties: A Review

**DOI:** 10.3390/molecules28186628

**Published:** 2023-09-14

**Authors:** Liping Jin, Chenpeng Ji, Shun Chen, Zhicong Song, Juntong Zhou, Kun Qian, Wenwen Guo

**Affiliations:** Key Laboratory of Eco-Textiles, Ministry of Education, College of Textile Science and Engineering, Jiangnan University, 1800 Lihu Avenue, Wuxi 214122, China; 6223016009@stu.jiangnan.edu.cn (L.J.); 6223017060@stu.jiangnan.edu.cn (C.J.); 6213011001@stu.jiangnan.edu.cn (S.C.); 1091210212@stu.jiangnan.edu.cn (Z.S.); 1091210214@stu.jiangnan.edu.cn (J.Z.); qiankun_8@163.com (K.Q.)

**Keywords:** flame retardant, antibacterial, functional textiles, finishing techniques

## Abstract

It is well known that bacterial infections and fire-hazards are potentially injurious in daily life. With the increased security awareness of life and properties as well as the improvement of living standards, there has been an increasing demand for multifunctional textiles with flame retardant and antibacterial properties, especially in the fields of home furnishing and medical protection. So far, various treatment methods, including the spray method, the dip-coating method, and the pad-dry-cure method, have been used to apply functional finishing agents onto fabrics to achieve the functionalization in the past exploration stage. Moreover, in addition to the traditional finishing technology, a number of novel technologies have emerged, such as layer-by-layer (LBL) deposition, the sol-gel process, and chemical grafting modification. In addition, some natural biomasses, including chitin, chitosan (CS), and several synthetic functional compounds that possess both flame-retardant and bacteriostatic properties, have also received extensive attention. Hence, this review focuses on introducing some commonly used finishing technologies and flame retardant/antibacterial agents. At the same time, the advantages and disadvantages of different methods and materials were summarized, which will contribute to future research and promote the development and progress of the industry.

## 1. Introduction

Textiles, as a common product made of fiber materials, have been extensively utilized in all aspects of our daily lives and industries. However, due to the general tendency of textile fiber materials to burn and cause fires, it can result in damage to property and even human life. Therefore, the application expansion of textiles is highly limited. For decades, tremendous efforts have been made to enhance the flame retardancy of textiles by incorporating flame retardants into the fiber matrix or directly modifying the surface of textiles.

Surface modification is the most commonly used technique thanks to its simplicity and ease of operation for both synthetic and natural fabrics [1]. A wide variety of flame retardants are used to endow textiles with good flame retardancy, mainly involving inorganic or organic flame retardants. The common inorganic flame retardants, mainly inorganic phosphorus-containing, boron-containing, zinc-containing, iron-containing, and carbon-based materials, and the frequently used organic flame-retardants such as halogenated, phosphorus-containing, nitrogen-containing, and silicone-containing flame retardants [2,3,4]. As early-stage commercial flame-retardants, halogenated compounds perform outstanding functions by releasing halogen radicals to eliminate reactive radicals during combustion [2]. Unfortunately, it has been abandoned in its actual application since its severe toxicity to the environment and human safety [5]. In response to this challenge, halogen-free flame retardants have emerged, and phosphorus-containing flame retardants stand out. The high flame retardancy is due to the promotion of the substrate to form a char layer, which isolates the transfer of heat and combustible gases, thus preventing further combustion of the substrate [2,3]. In addition, the silicone-containing flame retardants usually form a vitrified layer on the polymer surface during combustion, which effectively hinders the transfer of oxygen, heat, and mass and reduces the flammability of the polymer [6,7]. In contrast, nitrogen-containing flame retardants release noncombustible gases to dilute combustible gases such as oxygen during combustion, and the flame retardant effect is relatively poor but friendly to the environment [8]. To effectively improve the flame retardant efficiency, the combination of different elements with flame retardant properties can provide synergistic flame retardant effects and impart additional thermal stability and mechanical properties to the composites. In general, the study of synergistic flame retardancy has gradually become the emphasis of flame retardancy research in recent years [9].

Since the outbreak of COVID-19 in 2019, its prevalence has had a terribly negative impact on human health and caused enormous panic in the public. Against this background, the demand for medical protective equipment and anti-bacterial textiles is on the rise. As we all know, textiles have been protecting humans from external environmental harm for a long time. However, some textiles can also serve as breeding grounds or carriers for bacteria and viruses due to their hygroscopicity [10], which may lead to the risk of inflammation, disease, and even death in the human body. Therefore, the antibacterial treatment of some fabrics has become extremely urgent and has received widespread attention from scholars. Notably, the surface treatment of fabrics with antibacterial agents is the most commonly used strategy, which is similar to the flame retardant surface treatment of fabrics. In addition, antibacterial agents are mainly divided into three categories: inorganic antibacterial agents (metal and oxide nanoparticles (NPs), carbon-based antibacterial materials along with their composites), organic antibacterial agents (quaternary ammonium salts, guanidine, halogenated amines, phenols, etc.), and natural antibacterial agents (chitin, CS), and then the antibacterial or bactericidal effect of the antibacterial substances is mainly exerted by either directly contacting the bacterial surface or releasing the antibacterial moiety onto the substrate [11,12]. With the emergence of inorganic and organic antibacterial agents, the industrial application of antibacterial products continues to deepen, and they are playing an irreplaceable role in the functional textile industry. 

With the improvement of living conditions and the development of science and technology, the demand for multifunctional textiles in the market has grown in recent years. In particular, there is a large demand for flame retardant and antibacterial textiles in areas such as household products and medical protection. In order to develop novel functional fabrics with both flame-retardant and antibacterial properties, the relevant specific functional reagents and treatment methods have attracted considerable attention. Flame retardant and antibacterial dual-functional fabrics are usually achieved via a two-step or one-step method. The two-step method generally achieves the superposition of dual functions by introducing flame retardant and bacterial inhibitors in steps, which has the advantages of simplicity and wide applicability. However, there is a problem that some flame retardants or antibacterial agents may interact with each other, thus causing functional antagonism that is not conducive to simultaneously imparting excellent flame retardant or antibacterial properties to a fabric when the functional coating is applied in a stack. One-step methods normally treat fabrics by using synthetic agents with both flame retardant and antibacterial functions. However, the design of such multifunctional compounds is often difficult, and the synthesis process is complex. Therefore, it is a tremendous challenge to find the best processes and functional materials in the current direction of multifunctional textile research.

It is worth nothing that the specific finishing techniques have a pivotal influence on the processing efficiency and overall performance of the fabric. Then the traditional after-finishing technology mainly involves impregnation, padding, spraying, and pad-dry-cure techniques. These methods often do not require additional physical and chemical reactions but instead directly use functional solutions to treat the surface of fabrics, which have the advantages of being simple, effective, and easy to operate, but have the drawback of poor durability. In recent years, some promising environmentally friendly strategies have gradually attracted attention, such as LBL deposition, which usually uses deionized water as the solvent and positive and negative electrolytes as functional treatment agents [13]. Similarly, the sol-gel process is another environmentally friendly strategy that is favorable for the construction of functional surfaces for textile fibers by depositing thin organic-inorganic hybrid sol-gel films. In addition, this method has been selected as a simple and effective method to form a multifunctional protective coating since two or more siloxane precursors with different organic functions could be applied simultaneously [14,15]. Compared with the aforementioned techniques, chemical grafting modification exhibits unprecedented durability due to the stronger chemical bond linkage between functional agents and substrates [16]. Specifically, flame-retardant and antibacterial coatings are composed of inorganic nanomaterials, metal ions, or metal oxides. In situ modification technology has been extensively used for the construction of these coatings. 

There is no systematic summary of research on flame-retardant antibacterial fabrics, especially the accompanying finishing techniques. This paper reviews the latest research results about the flame-retardant and antibacterial functional finishing of textiles over the past decade. Furthermore, it explores the nascent finishing agents as well as the adaptable post-finishing technique. Simultaneously, this review also discusses the advantages, disadvantages, and application scope of these techniques and briefly introduces the development of green environmental technologies.

## 2. Methods and Standards for Flame Retardant and Antibacterial Tests

### 2.1. Methods and Standards for Flame Retardant Tests

#### 2.1.1. Cone Calorimetry

According to ASTM Standard E1354, the flame retardancy of the material was evaluated by the heat value of combustion released by subjecting a 10 × 10 cm^2^ sample to a radiant heat stream (≤100 kWm^−2^) in the presence of an ignition source [17].

This paper mainly provides information such as the peak heat release rate (HRR), total heat release (THR), and peak heat release rate (PHRR) [18]. In general, the lower the value, the better the flame-retardant effect.

#### 2.1.2. Limiting Oxygen Index (LOI) 

According to ISO Standard 4589 and GB/T Standard 5454-1997, the lowest oxygen concentration to support the combustion of the material (limiting oxygen index) was obtained by igniting the tip of a vertically fixed sample (~7 × 15 cm^2^), accompanied by a continuously decreasing oxygen concentration until the flame was extinguished.

A high oxygen index indicates that the material is not easily combustible, while a low oxygen index indicates that the material is easily combustible. Flammable Materials: <22%; Combustible Materials: 22~27%; Refractory Materials: >27%.

#### 2.1.3. UL-94/Vertical Burning Test

According to the UL-94V standard, ASTM standard D3801, GB/T standard 17591-2006, and GB/T standard 5455-2014, the sample size was about 300 × 70 mm, which was exposed to a vertical flame for 12 s once or twice. Time to ignition, afterflame, afterglow, calculated residual mass, etc. were obtained.

There are two hierarchies that can be used for assessment: Firstly, V-0 (best grade), V-1 or V-2, wherein, V-2: after two 10 s combustion tests on the sample, the flame is extinguished within 60 s and combustibles are allowed to fall off; V-1: after two 10 s combustion tests on the sample, the flame is extinguished within 60 s and no combustibles can fall off; V-0: after two 10 s combustion tests on the sample, the flame is extinguished within 30 s and no combustibles can fall off [19]. Secondly, B1, B2, wherein, B1: time to ignition ≤ 5 s, time to deflagration ≤ 5 s, length of damage ≤ 150 mm; B2: time to ignition, time to deflagration ≤ 15 s, length of damage ≤ 200 mm.

### 2.2. Methods and Standards forAntibacterial Tests

#### 2.2.1. The Shake Flask Method

According to GB/T standard 20944.3-2008, the rate of bacterial inhibition was obtained by determining the concentration of surviving bacteria in the specimen and control samples, respectively, after a period of shaking.

A sample has an antimicrobial effect if it inhibits *Staphylococcus aureus* and *Escherichia coli* by ≥70%.

#### 2.2.2. The Inhibition Zone Method

According to SNV standard 195920–1992, the fabric with a diameter of 2 cm was placed on the agar medium and incubated at 37 °C for 24 h. The clear media around the disc indicates that the bacterial growth around the samples was inhibited, and the diameter of the area was measured to assess the antimicrobial activity of the samples.

In general, if the diameter of the suppression ring is less than or equal to 7 mm, it is judged as having no suppression; if the diameter of the suppression ring is greater than 7 mm, it is judged as having suppression.

#### 2.2.3. AATCC Test Method 

According to ASTM standard 147-2004, successive parallel lines were drawn on the agar medium inoculated with bacterial solution, and the sample was uniformly attached to the parallel stripes. After incubation for a period of time, clean areas will appear on the agar surface at the delineated areas due to the interruption of bacterial reproduction. The antibacterial activity against bacteria was examined by measuring the average clear inhibition zone using the relevant equation.

The size of the zone of inhibition cannot be used as a quantitative assessment of antimicrobial activity. However, antimicrobial-treated materials are comprehensively evaluated by comparing them to untreated materials and to sample materials with known inhibitory activity and including observations of the zone of inhibition.

## 3. Recent Advances in Multifunctional Textiles with Flame Retardant and Antibacterial Properties

The functional finishing of traditional textiles is mainly divided into two strategies. One is to mix the functional agents with textile raw materials and prepare functional textiles after the spinning process. This method has the advantage of good washing durability, but the cost is relatively high and usually has a significant influence on the mechanical properties of textile fibers. The other method is to perform surface modification treatments on textiles. Currently, surface modification technology is commonly used for the fabrication of functional fabrics such as flame retardant, antibacterial, and hydrophobic fabrics, which is a simple, convenient, and efficient way to endow traditional fibers/fabrics with specific functions. In the process of functional finishing, such as flame retardancy and bacteriostasis, the finishing techniques have a great influence on the final performance of the fabrics. Generally speaking, the finishing technology for fibers/fabrics mainly includes traditional finishing methods such as dip-coating [20] and spraying techniques [15] and some novel finishing strategies, including chemical grafting modification [21,22], layer-by-layer self-assembly [23,24], sol gel [25,26], and in situ deposition [27].

Ulteriorly, the traditional finishing technology of fabrics mainly includes the traditional impregnation method, pad-dry-cure method, coating method, and spray method. These methods have low finishing costs but unsatisfactory washability, and long-term use of flame-retardant effects will be affected by water, light, and other conditions. Presently, various surface-modifying technologies, such as the sol-gel method, nanoparticle adsorption, layer-by-layer self-assembly method, plasma treatment, and the graft copolymerization modification method, have been utilized for preparing flame-retardant, anti-bacterial, hydrophobic, UV-resistant, self-cleaning, multi-functional textiles on the basis of synergistic flame-retardant technology.

### 3.1. The Traditional Finishing Techniques

#### 3.1.1. The Spray Method

The spraying method is one of the traditional flame-retardant finishing technologies. The finishing agents were dissolved into a certain solvent and then introduced onto the surface of fabric by simple spraying, which easily forms a thin functional coating on fabric surfaces [28]. For instance, Attia et al. developed the novel nanocomposites (DPHM-Ag NP) based on diphosphate malonate as organic phosphates and silver NPs, and then the nanocomposites coatings were sprayed on the surface of polyester (PS) and cotton-polyester (CB) blend fabrics. The treated fabric meets the standards of high-class flame-retardant textiles with a 0 mm/min rate of burning. Furthermore, the antibacterial properties were enhanced with the clear bacterial inhibition zone reaching 4.48 mm for *Staphylococcus aureus* (*S. aureus*) [29].

#### 3.1.2. The Dip-Coating Method

**Figure 1:**
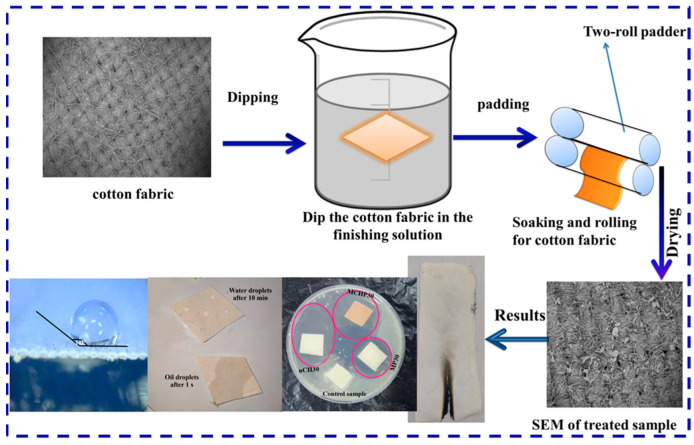
A schematic diagram for preparing cotton fabrics coated with CS-based flame retardant coatings [33] (Copyright 2022, Elsevier).

Dip-coating is a finishing method that involves immersing fabrics in a functional agent solution and is accompanied by a drying treatment [30]. This method is simple to operate; the crosslinking reaction between the fabric and the flame retardant is weak during the process of finishing, and most of the flame retardant is just attached to the surface of the fabric, so the durability of the flame-retardant fabric is generally poor. To achieve the flame-retardant antibacterial properties of textiles, a novel agent (tetramethylcyclosiloxyl-piperazin) tetra guanidine (GNCTSi) was designed and successfully applied to form a functional coating on the surface of cotton fabrics. The treated cotton fabrics have enhanced properties, with LOI reaching 30.1% and char length remaining at 6.5 cm after burning. To a certain extent, the coating improves washing durability and thus has less impact on the flame retardancy of cotton fabrics. Furthermore, it also exhibits improved antibacterial effects with inhibition zones of 2.5 mm and 2.3 mm against *Escherichia coli* (*E. coli*) and *S. aureus*, respectively [31]. In addition, Atousa et al. prepared a suspension with ZrO_2_ NPs along with cetyltrimethylammonium bromide (CTAB), maleic acid (MA), sodium hypophosphite (SHP), and urea by using an impregnation bath. MA was used as a cross-linking agent, while SHP acted as the catalyst to stabilize NPs on the fabric surface and prevent fabric from creasing. In the end, the test results showed improved flame-retardant properties, antibacterial activities, and self-cleaning properties of the treated cotton fabrics [32]. In order to enhance the coating’s durability, some polymeric coatings, such as polyvinyl alcohol (PVA) and polyurethane (PU), were used as binder for flame retardant components. In the work of Ghada et al., nano chitosan (n CS), melamine phosphate (MP), and melamine salt of CS phosphate (MCSP) were prepared and then mixed with PVA to construct PVA/MCSP and PVA/n CS/MP coatings on cotton fabrics by the dip-coating method (as shown in Figure 1). The PVA/MCSP30 coating displayed the optimum flame resistance with self-extinguished behavior and a very high LOI of 58.2%, while the LOI for the original fabric was only 17.2%. In addition, it also exhibited good coating durability as well as better antibacterial properties for both *E. coli* (inhibition zone diameter of 27.6 mm) and *S. aureus* (inhibition zone diameter of 30.5 mm) [33]. Similarly, the condensed tannin extract from Dioscorea cirrhosa tubers was also used as the functional agent for silk fabric, and the treated fabric has good antibacterial properties and flame retardancy [34].

#### 3.1.3. The Pad-Dry-Cure Method

The pad-dry-cure method treats the fabric by padding, drying, and curing it after it has absorbed enough functional solution [35]. In these processes, high-temperature drying makes flame-retardant or antibacterial functional materials chemically cross-linked with fabrics to obtain durable flame retardancy. Ahmed et al. first fabricated blended fabrics with different compositions, weaving structures, grams per square meter, thicknesses, and thread densities. In the following step, a three-dimensional tetrakis (hydroxymethyl) phosphonium chloride (THPC)-urea polymer coating was synthesized and deposited onto the blended fabrics. The finished fabric showed excellent antibacterial properties (99.9%) and excellent flame retardancy (LOI~36.8%); additionally, it also possessed superior water repellence properties (151.5°) [36]. Singh et al. prepared a thyme oil-embedded functional microcapsule via in situ synthesis of CS phosphate as the shell material, and then the microcapsules were introduced onto linen fabrics via the pad-dry method. The finished fabric presented excellent antibacterial properties (>98%), flame retardancy (LOI > 28), antioxidant activity (96%), mosquito repellency (100%), and an excellent fragrance. Moreover, the functional properties were durable after at least 20 washes [37].

The pad-dry-cure method was also applied to deposit nanocomposite coatings on fabrics. The TiO_2_ NPs were prepared by the sol-gel method using titanium tetraisopropoxide. The development of nano TiO_2_ onto cotton fabric was accomplished when nano TiO_2_ was coated onto cotton fabric by the traditional pad-dry-cure method in the presence of polycarboxylic acid [1,2,3,4-butane tetracarboxylic acid], SHP, and CS phosphate. The results confirmed that 1,2,3,4-butane tetracarboxylic acid, TiO_2_, and CS phosphate are helpful in increasing the flame resistance and antibacterial properties of cotton fabrics [38]. Similarly, Dhineshbabu et al. prepared colloidal methyl silicate and MgO nanoparticle-embedded methyl silicate solutions through the sol-gel method. Subsequently, cotton fabrics were separately modified with silica and MgO/methyl silicate composites via an optimized pad-dry-cure method. The MgO/methyl silicate composite-coated fabrics showed enhanced burning performance, significant water-repellent properties, and better antibacterial activity against *S. aureus* and *E. coli* than methyl silicate-coated and uncoated fabrics [39]. In another work, an equimolar sol mixture of the precursors P, P-diphenyl-N-(3-(trimethoxysilyl)propyl) phosphinic amide (SiP) and 1H,1H,2H,2H-perfluorooctyltriethoxysilane (SiF) was employed to form a two-component sol-gel inorganic–organic hybrid coating on the fabric by the pad-dry-cure method. It leads to good flame retardancy and antibacterial properties, with an inhibition rate of 92.9% against *E. coli* and 80.4% against *S. aureus* [40].

### 3.2. LBL Self-Assembly Technology

Whether in textile flame retardant finishing or other fields, LBL self-assembly technology has been widely used because of its simple operation, ease of control, and environmental friendliness [41]. It is a simple and versatile technology for preparing multifunctional coatings. These coatings are formed by repeatedly depositing alternate layers of oppositely charged materials, which experience attraction and undergo self-regulation within individual layers by electrostatic action. LBL self-assembly can be uniformly coated on the surface of textiles to achieve flame retardant, antibacterial, and other multifunctional properties, which have been applied to PS, cotton, polyamide, and silk fabrics [42]. At present, the commonly used positively charged compounds mainly include CS, polyetherimide (PEI), DL-arginine (DL), etc. The negatively charged electrolytes mainly include phytic acid (PA), sodium alginate (SA), ammonium polyphosphate (APP), and so on. Except for flame retardancy, some of these charged materials can also endow base materials with antibacterial properties, while others need to be combined with antibacterial materials to achieve multi-functionalization.

**Figure 2:**
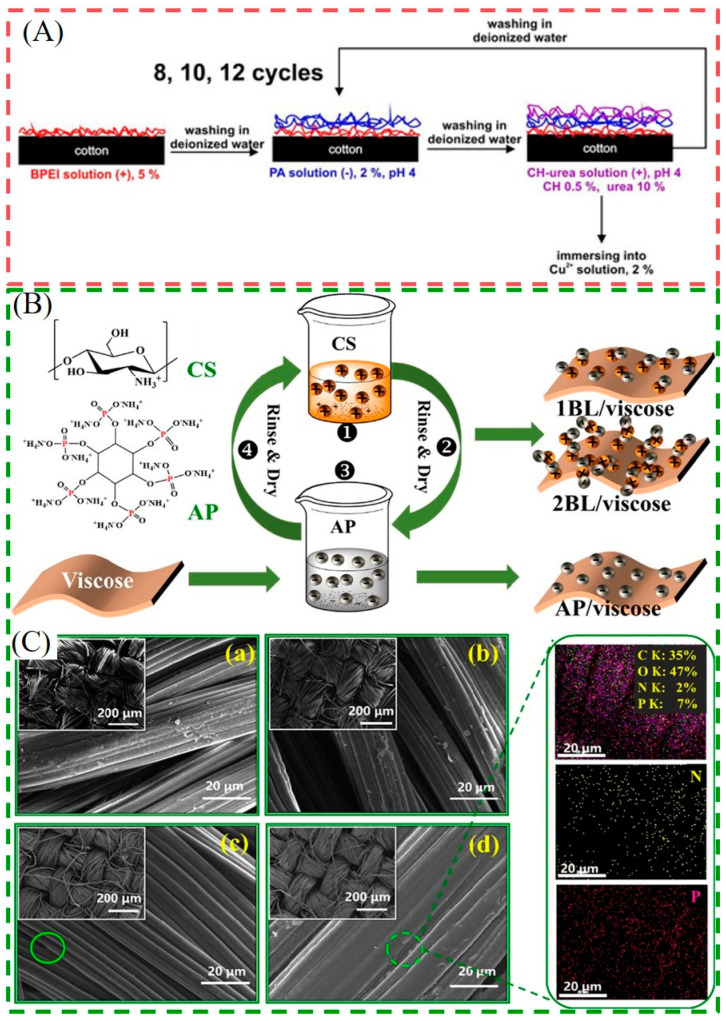
(**A**) The LBL deposition process of FR/antimicrobial nanocoating on cotton [13] (Copyright 2020, Elsevier). (**B**) The preparation process of 1BL/viscose, 2BL/viscose, and AP/viscose. (**C**) SEM pictures (×200 and ×4000) of the pure and coated viscose fabrics: (**a**) viscose, (**b**) AP/viscose, (**c**) 1BL/viscose, (**d**) 2BL/viscose, and EDS mappings of 2BL/viscose [45] (Copyright 2021, Elsevier).

Herein, the bio-based materials PA and CS are often selected to impart flame retardant as well as antibacterial properties to fabrics through LBL technology. PA and its salts, such as ammonium phytate (AP), are generally composed of a large number of phosphoric acid groups and ammonium ions. They can catalyze the degradation of fabric substrate to form more char residues and release incombustible gases such as NH_3_ or N_2_ to dilute combustible gases during combustion. Therefore, the flame-retardant properties of fabrics in both condensed and gaseous phases are ultimately improved [1,43]. CS contains NH_2_ groups that can generate -NH^3+^ groups under acidic conditions, so it applies as a positive charge in LBL self-assembly. Moreover, the -NH^3+^ group can destroy the cell wall or interfere with the normal physiological activities of cells, thus exerting both flame retardant and bacteriostatic effects [44]. Liu et al. deposited CS and AP on cotton fabric to manufacture fully bio-based flame-retardant and antibacterial cotton fabrics using LBL technology. The modified textile with a low weight gain (8 wt%) performed perfect self-extinguishing behavior in the vertical burning test. Moreover, the CS/AP coating has an effective antimicrobial rate of 99.83% against E. coli, which mainly depends on the introduction of CS [44]. In their other work, a fully bio-based CS/AP coating was also applied to a viscose fabric (as shown in Figure 2B,C)). The 2BL/Viscose showed sharp improvements in thermal stability in the higher temperature zone, accompanied by a LOI of 29% and self-extinguishing behavior in the combustion test. Additionally, 2BL/Viscose possessed a high bacteriostatic function of 99.99% for both *E. coli* and *S. aureus* [45]. However, the antibacterial ability of the system using PA/CS alone is limited and generally requires synergistic use with other antibacterial agents. In order to enhance the antimicrobial ability and improve the broad-spectrum antibacterial effect, it is necessary to introduce other antibacterial agents into the CS/AP coatings. For instance, Eva et al. soaked the LBL-treated samples in a 2% Cu^2+^ solution after depositing PA and CS-urea on cotton. The VFT results showed that 12 BL of PA/CS-urea-Cu^2+^ could stop the burning flame, and the PHRR and THR were reduced by 61% and 54%, respectively. Moreover, for the antibacterial test, 100% reduction of *S. aureus* and Klebsiella pneumoniae can be achieved (as shown in Figure 2A) [13].

Arginine, a kind of novel renewable material, has an alkaline cationic chemical group that possesses high antibacterial properties [46]. In addition, non-combustible gases are released during combustion, acting as a gas-phase flame retardant [47]. Recently, many researchers have proven that the combination of arginine and CS can have a good inhibitory effect on cell growth and hence exert an antibacterial function. Moreover, arginine-functionalized CS also exhibited good flame retardancy and smoke suppression [48,49,50]. Except for CS, PA can also chelate with the ammonium ions of DL through hydrogen bonds, and they were deposited on CB fabric using LBL assembly. The finishing CT fabric with 20 bilayers showed enhanced fire safety and an efficient inhibition diameter of 4.0 mm against *S. aureus* compared to the untreated CT fabric (0 mm) [51]. In addition, bio-based riboflavin sodium phosphate (vitamin B2, VB2) is an anionic phosphorus-containing compound that has been extensively used in disease treatment. The negatively charged VB2 can cooperate with CS to be used as the LBL assembly agents for the modification of silk fabric, and the prepared colored silk fabric with 10 assembly bilayers exhibited great flame retardancy and antibacterial performance (>90% inhibition rate against both *S. aureus* and *E. coli*). The employment of LBL technology to prepare fully bio-based flame-retardant and antibacterial coatings follows the concept of environmentally friendly development and has been widely used for the functional modification of fabrics [52].

Among numerous antibacterial agents, N-halamines have attracted extensive attention due to their broad-spectrum antibacterial activity, long-term efficacy, and renewability [53,54]. With the great demand for multifunctional materials, a novel nitrogen/silicon-containing N-halamine cationic polymer (PCQS) containing both flame-retardant and antibacterial components has been designed and used as the positive electrolyte, which then interacts with negatively charged PA molecules to coat cotton fabric. The cotton-PEI/(PCQS/PA)_30_-Cl exhibited an increased LOI of 28.5% with a lower char length of 7.9 cm in the vertical flammability test. In addition, the treated fabric could inactivate 6.01 logs of *S. aureus* and 6.00 logs of *E. coli* within 1 min of contact time, demonstrating effective antimicrobial activity [55].

### 3.3. Chemical Grafting Modification

Chemical grafting modification is a technique that introduces functional groups to fibers or fabrics by forming covalent bonds. Flame retardant and antibacterial functionalization can be realized via the chemical grafting technique, which generally shows higher durability than other finishing methods [56]. As a typical scale inhibitor, diethylene triamine penta methylene phosphonic acid (DTPMPA) is rich in N and P elements and has become a potential flame retardant. In addition, the phosphonic acid groups in the structure of DTPMPA provide sufficient binding sites that can easily chelate with metal ions, such as silver ions. The lyocell fabric with flame-retardant properties (FR-lyocell) has been accomplished by grafting a novel flame-retardant ammonium salt of diethylene triamine penta methylene phosphonic acid (ADTPMPA). Subsequently, the FR-lyocell was further treated with Ag nanoparticles (Ag NPs) to develop antibacterial properties. The flame retardant and antibacterial lyocell fabric (FRAg-lyocell) exhibits unbelievable flame retardancy (LOI of 44.8%) and good washing durability (LOI has still been maintained at 31.3% after nearly 20 laundering cycles). Moreover, pHRR and THR values were suppressed effectively. At the same time, it also possesses excellent antimicrobial ability against both *S. aureus* and *E. coli* [56]. Xu et al. synthesized a water-soluble N-halamine precursor based on s-triazine (TIAPC) by introducing iminodiacetic acid. After grafting modification with TIAPC, the treated cotton fabric was then chlorinated with NaClO solution and chelated with metal Al^3+^ ions. Cotton-TIAPC-Cl-Al presented a high-efficacy and rapid bactericidal effect against *S. aureus* and *E. coli*, with 100% bacterial reduction in 1 min. In addition, the hydrophobic property of cotton-TIAPC-Cl-Al was greatly improved after chlorination. However, it cannot self-extinguish in the vertical burning test, implying limited flame retardancy [57].

**Figure 3:**
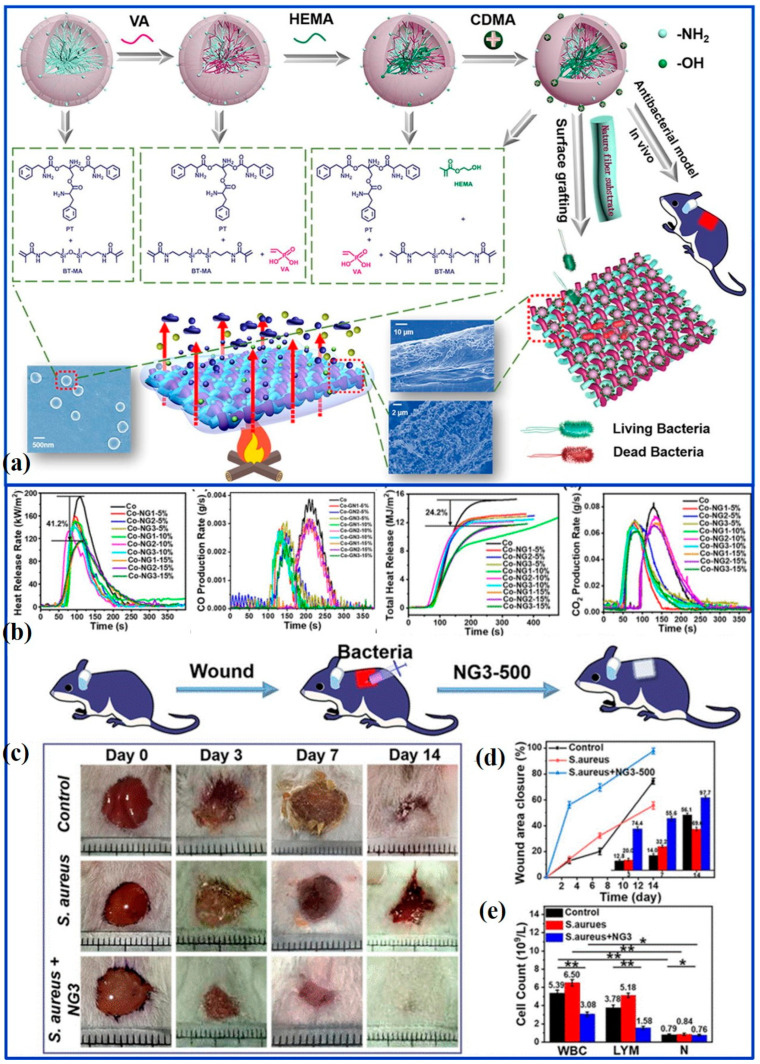
(**a**) Graphical abstract. (**b**) Cone calorimeter curves of Co-NG1, Co-NG2, and Co-NG3. (**c**) Photographs of wounds treated by the control, *S. aureus* and *S. aureus* + NG3-500 nanogels (the minimum scale is 1 mm). (**d**) Assessment of the wound size reduction. (**e**) Expression level of WBC, LYM, and N in the blood of mice wounds after 3 d of treatment. Results are presented as mean ± standard deviation, and * indicates a significant difference (*p* < 0.05); ** indicates a significant difference compared with all other conditions (*p* < 0.01) [16] (Copyright 2021, Elsevier).

Recently, nanogels, with large specific surface areas and higher functional efficiency, have been widely exploited as antimicrobial materials [58]. However, to date, there are very few reports in the literature on the use of nanogels for antimicrobial and flame retardant applications. In the work of Li et al., novel nanogels (NG3) were synthesized by Michael addition, which contain both the flame retardant elements of phosphorus, nitrogen, and silicon and the antibacterial component of N, N’-dimethyl-N-(3-(trimethoxysilyl) propyl) dodecane-1-chloroamine. By grafting on the cotton fibers and fabrics, the treated cotton fabric has self-extinguishing behavior, implying improved fire safety. Because NG3 easily destroys cell membranes and causes cell lysis, the grafted cotton fabrics can eliminate nearly 99% of bacteria for both *S. aureus* and *E. coli*. In addition, the NG3 with good biocompatibility and antibacterial properties plays a positive role in preventing wound infections, and anti-infection experiments of healing efficiency reaching 97.7% after 14 days’ treatment have confirmed it. More importantly, functional cotton fabrics modified with nanogels exhibit relatively low mechanical and comfort properties (Figure 3) [16]. In another work, a binary mixture of acrylonitrile and 4-vinyl pyridine under the condition of ceric ammonium nitrate as initiator was copolymerized and grafted on the cotton fabrics by chemical induction. Taking advantage of the flame-retardant properties of synergistic nitrogen and phosphorus elements, the modified cotton fabrics have self-extinguishing abilities with a slow propagation rate. Meanwhile, the treated samples possess excellent antibacterial properties, with 41~96% antibacterial activity [59].

The guanidyl-based organic compounds can be used as antibacterial agents, which have the advantages of nontoxicity, high-temperature resistance, outstanding antibacterial effects, and long action periods. Commonly, the nitrogen-containing guanidyl group in the guanidyl-based antibacterial agent also exerts a better flame-retardant effect that can be combined with a phosphorus-containing group to form a phosphorus-nitrogen synergistic flame-retardant and antibacterial agent. Two novel and efficient antibacterial and flame-retardant guanidine-based compounds, N, N-di (ethyl phosphate) biguanide (DPG) and mono chlorotriazine triethyl phosphite guanidine (MCTPG), were successfully synthesized and then grafted onto cotton fabric by generating covalent bonds. The treated cotton fabric obtained good flame retardancy and antibacterial properties; the former DPG-treated cotton fabric obtained an LOI of 31.2%; and the antibacterial ratios of *S. aureus* and *E. coli* were 96.4% and 99.2%, respectively. The latter MCTPG-treated cotton fabric gained a LOI value of 31.2%, and the char length decreased to 8.5 cm. In addition, its inhibition zone base for *E. coli* and *S. aureus* reached 2.9 mm and 2.8 mm, respectively [10,60].

### 3.4. In Situ Deposition of Inorganic Metal Materials

In recent years, due to its excellent thermal and catalytic properties. Additionally, metal materials have excellent antibacterial properties with free radical capture abilities that have a wide application prospect in the functional fields of antibacterial and UV resistance. These metal materials were deposited on the surface of fabrics by direct reduction or synthesis [61]. 

#### 3.4.1. In Situ Deposition of Neat Metallic Oxide

The biomedical applications of NPs, especially metal oxides, have attracted great interest. ZnO NPs are the most famous type of NPs that inhibit the growth of Escherichia coli. Moreover, ZnO NPs with low cost, non-toxicity, and recyclability also acted as efficient photocatalysts. However, their easy deactivation and low acid resistance severely limit the development of ZnO NPs. To solve the problem, Bahare et al. synthesized ZnO@SiO2 NPs and coated them on the PET fabric by applying zinc acetate and sodium silicate as two precursors in an aqueous ammonia solution at 90 °C. The silica-supported ZnO improved these problems by limiting the size of the NPs (approximately 28.29 mm) and protecting them from acid solution corrosion. The treated samples have enhanced anti-dripping properties because of the inherent thermal resistance of Si. Furthermore, the treated PET fabrics eradicated almost 100% of *E. coli* [62]. However, some synthetic fibers, such as PS fibers, lack chemically active groups and have compact structures, which makes it difficult to absorb and carry functional reagents. In order to realize compatibility between these fiber products and functional components, the solvent crazing technique is applied to these fiber structures [63].

#### 3.4.2. In Situ Deposition Assisted by Polymer Coatings

Unfortunately, metal oxides often have insufficient adhesion to fabrics when used alone. Polymer coating is helpful to improve the adhesion of metal compounds to the surface of fabrics. Commonly, pyrrole and aniline are used to prepare polymer coatings on fabrics that could provide active sites for metallic or other inorganic materials. Mahmoud et al. produced a polypyrrole-silver composite (Ppy-Ag) coating on the cotton/PS substrate through vapor phase polymerization (VPP) and redox reactions [64]. Polypyrrole can act as an effective stabilizer for Ag NP to solve the instability problem of treated fabrics after washing steps. The coated textile displayed an inhibition zone diameter of 25 mm and 28 mm for *E. coli* and *S. aureus*, respectively, verifying a supreme antibacterial property. It also had superior conductivity features with a low electrical resistance of 0.0218 kΩ. Furthermore, the treated fabrics show good washing fastness, implying improved stability of silver-containing coatings for textiles [64]. Polyaniline (PANI), a popular conducting polymer, has been regarded as a flame retardant for polymers due to its better char-forming ability. Cai et al. fabricated a PANI @ TS-silk fabric electrode that exhibits good charging and discharging cycle stability and high area-specific capacitance. Furthermore, the treated fabric electrode has good flame retardance and excellent antibacterial properties (99%) [65]. In another work, polyaniline was polymerized to form polyaniline chains on the surface of nanotubes in the presence of dispersed halloysite nanotubes. The polyaniline chains were decorated onto the fabric successfully due to the nanotubes aligned on the fabric’s surface. Compared with the untreated fabric, the burning rate of the coated fabric decreased by 72%, and the clear antibacterial inhibition zone was recorded at 6 mm. Furthermore, the tensile strength of coated textile fabrics was maintained due to the alignment of nanotubes on the surface of the fabrics [66].

#### 3.4.3. In Situ Deposition Assisted by Complexation Reaction

Many organic compound molecules (ions) containing unsaturated or active groups such as amino, carboxyl, and hydroxyl groups are prone to interact with metal ions, and organometallic complexes are usually obtained through certain coordination, complexation, and redox reactions. At present, this method is widely used in fabric functional finishing. Owing to the abundant nitrogen source, guanidine salts (e.g., guanidine carbonate, nitrate, and phosphate) exhibited intriguing flame retardancy. These compounds are able to improve the flame retardancy of the polymer matrix and promote the formation of the carbonized layer, which can act as a physical barrier. Guanazole, a low-cost compound with the chemical structure of 3,5-diamino-1,2,4-triazole, can coordinate with metal centers, so it has become an excellent flame retardant ligand. Hu and Wang et al. formed guanazole-zinc and guanazole-silver in aqueous solutions and then deposited them on cotton fabric surfaces by a dipping process. As expected, the cotton fabrics modified with guanazole-zinc and guanazole-silver exhibit outstanding flame retardancy with 29.5% and 27.5% LOI, respectively. Additionally, the samples possess self-extinguished behavior after removing the igniter during the vertical burning test and have reached the UL-94 V-0 level of flame retardant after the vertical burning test. In the micro-scale combustion calorimeter test, the HRR of guanazole-zinc and guanazole-silver modified cotton fabrics, respectively, reached 64.4% and 59.1%, while the THR of guanazole-zinc and guanazole-silver modified cotton fabrics reached 26.4% and 14.8%. More than this, the treated cotton fabrics also showed augmented antibacterial capacity against *S. aureus* and *E. coli*. Notably, the guanazole-silver-coated cotton fabrics also reflect the antifungal effect on Penicillium, Aspergillus niger, and Fusarium chlamydosporum [67].

**Figure 4:**
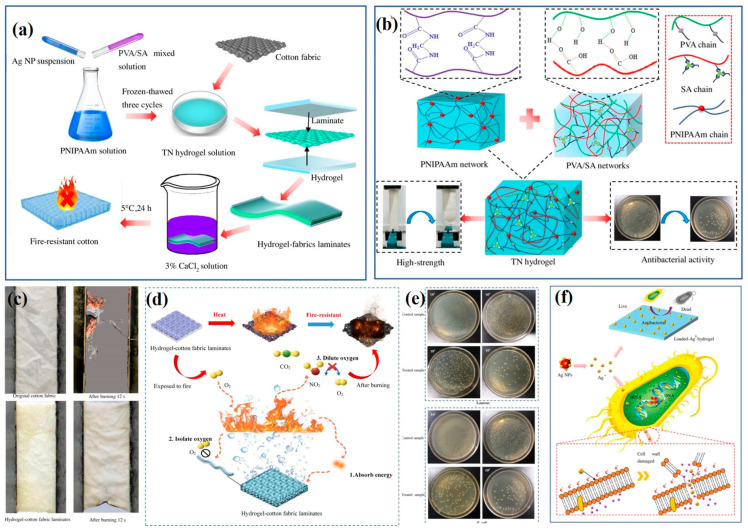
(**a**) Schematic diagram of the preparation of fire-resistant hydrogel-fabric laminates. (**b**) Interpenetrating polymer network structure and antibacterial activity of triple-network composite hydrogels. (**c**) Vertical fire-retardant testing of untreated cotton fabric and hydrogel–fabric laminates. (**d**) Schematic illustration of the mechanism of fire resistance of hydrogel-cotton fabric laminates. (**e**) the picture of bacteria recovered from the samples against *S. aureus* and *E. coli*. (**f**) schematic illustration of the antibacterial mechanism of Ag NPs [68] (Copyright 2021, Elsevier).

As we all know, water and fire are incompatible. However, it is difficult to directly apply water as a fire-resistant material due to its high mobility. Hydrogel polymers with water as the main component can be used as fire-retardant materials to form a fire-resistant layer and reduce water loss, thereby improving the flame-retardancy of the coated fabric. Therefore, in the work of Yu et al., a novel fire-preventing triple-network (TN) hydrogel composed of poly (N-isopropylacrylamide) (PNIPAAm)/SA/PVA was prepared and laminated on cotton fabric, which was then put through the ionic coordination crosslinking process in CaCl_2_ solution to form a stable structure. During the process, Ag NPs were also embedded into the hydrogel. Compared to neat fabric, the hydrogel-fabric laminates were nearly undamaged after being exposed to fire for 12 s, which is attributed to energy absorption as the water in the hydrogel is heated and evaporates. At the same time, outstanding antibacterial functions (>96%) against *E. coli* and *S. aureus* were achieved. Moreover, the introduction of a hydrogel layer also improves the mechanical strength of fabrics. Thus, the results demonstrated that the TN hydrogel, as a fire-resistant polymer, has potential for life-saving (Figure 4) [68].

**Figure 5:**
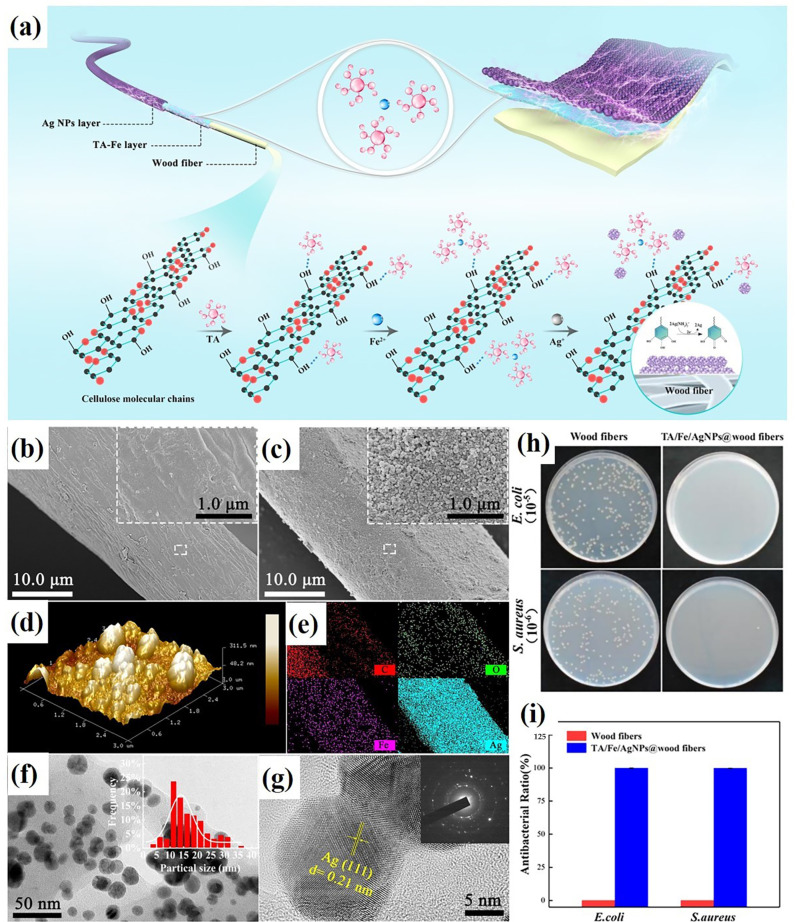
(**a**) Synthesis of TA/Fe/Ag NPs @ wood fibers. (**b**,**c**) SEM images of wood fibers: before and after modification. (**d**) AFM images of the modified fibers. (**e**) Element maps of C, O, and Ag for the sample shown. (**f**) TEM image of the modified fibers; inset: Ag NP size distribution. (**g**) HRTEM image of a single Ag NP showing the (111) lattice plane of Ag; inset: corresponding SAED image. (**h**) Impacts of TA/Fe/Ag NP modification on the antibacterial activity of wood fibers: Plate count tests show the effects of wood fibers with and without TA/Fe/ Ag NP modification on the growth of *E. coli* and *S. aureus*, including the appearance of counting plates and (**i**) antibacterial ratio [71] (Copyright 2021, Elsevier).

Metal phenolic networks (MPNs), which consist of a variety of phenolic compounds and metals, have been promising candidates for the surface functionalization of substrates. For decades, naturally occurring compounds and their derivatives as eco-friendly antibacterial and flame-retardant agents for fabrics have attracted extensive attention from scholars. Researchers [69,70] have applied MPNs on the surface of silk fabrics, wherein Zhang et al. combined tannic acid (TA) with ferrous ions to form FR and antibacterial materials and applied them to silk fabrics that possess durable increasing FR with a 27.5% LOI value and almost no decrease even after 20 washes. In the vertical burning test, the treated sample shows a damaged length of only 11.2 cm but 30.0 cm of the original. The antibacterial activity significantly increased from 22% to 95% and maintained over 90% of its properties even after 20 washes [69]. In another work, Cheng et al. extracted polyphenols under alkaline conditions to develop flame-retardant macromolecular polyphenols through oxidative polymerization. The silk was dyed with the aforementioned extracted natural dyes and post-mordanted with metallic salts. The results not only showed improved flame-retardant and antibacterial properties but also antioxidant behaviors, washing fastness, perspiration, and wet rubbing fastness [70]. The MPNs can be applied to wood fibers as well to solve the problem of limiting application caused by their poor flame retardancy and antibacterial behavior. In the study of Jiang et al., wood fibers were immersed in a single solution of TA and ferrous salt successively to form a TA-Fe-wood complex and then further modified with silver nanoparticles (Ag NPs) to structure an Ag NPs layer. The TA/Fe/Ag NPs@wood fibers were successfully prepared (as shown in Figure 5). In the test of cone calorimetry, the TA/Fe/Ag NPs@wood fibers displayed enhanced flame retardancy, with the peak heat release rate and the peak smoke production rate reducing by 71.5% and 56.5%, respectively. Not only that, it also increases antibacterial activity for both *E. coli* and *S. aureus*. At the same time, the problem of the matrix being darkened by MPNs was solved [71]. 

**Figure 6:**
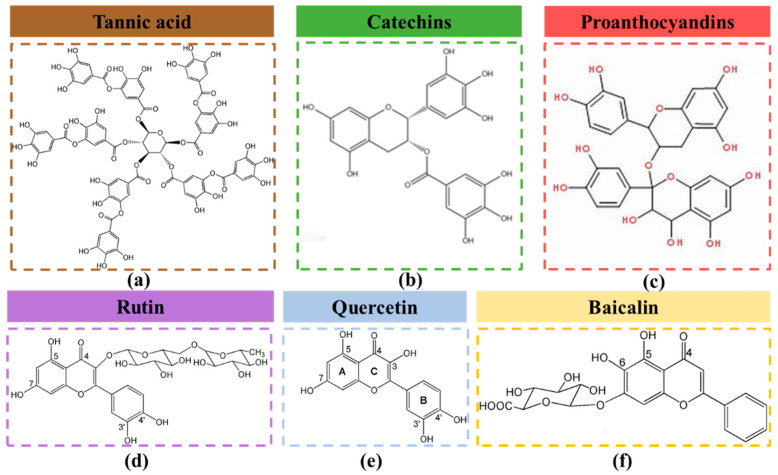
(**a**) Chemical structure of tannic acid [69] (Copyright 2020, Elsevier). (**b**) Chemical structure of Catechins. (**c**) Chemical structure of Proanthocyandins. (**d**–**f**) Chemical structures of three flavonoids [73] (Copyright 2019, Elsevier).

Apart from TA (Figure 6a), some other flavonoids, including Catechin (Figure 6b), Proanthocyandins (Figure 6c), Rutin (Figure 6d)), Quercetins (Figure 6f), Baicalin (Figure 6g), have attracted great attention in the fields of dyeing and functionalization of textiles simultaneously. Guo et al. applied grape seed proanthocyanidins (GSPs), which are a kind of recycled low-value byproduct rich in polyphenolic compounds, to the coloration of silk with a flame-retardant and antibacterial functionalized treatment. The dyed silk performs progressive flame retardancy due to the condensed phase flame retardancy mechanism of GSPs. In addition to enhancing the antibacterial properties effectively, washing, rubbing, perspiration, and light color fastness have also been improved to a certain extent [72]. Three flavonoids (baicalin, quercetin, and rutin) have been utilized in silk fabrics under the action of two metal salts (ferrous sulfate and titanium sulfate) mordanting as well. The results of the vertical burning test indicate improved flame-retardancy (the detailed data are shown in Table 1) and smoke suppression due to the good char formation ability of the silk fabrics in the process of combustion [73].

### 3.5. Sol-Gel Method

**Figure 7:**
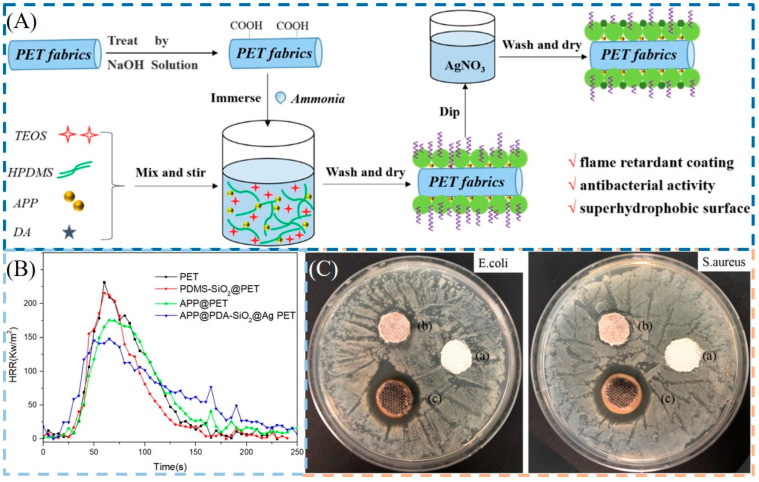
(**A**) Schematic illustration of the fabrication process for APP @ SiO_2_-PDA @ Ag PET fabrics. (**B**) HRR curves of the pristine and coated PET fabrics. (**C**) (**a**) Antibacterial activities of the pristine PET fabrics; (**b**) APP @ SiO_2_-PDA PET fabrics; and (**c**) APP @ SiO_2_-PDA @ Ag PET fabrics [76]. [Copyright 2021 Elsevier].

The sol-gel technique involves hydrolysis and condensation reactions using siloxane or metal alkoxide as precursors. First, a sol system is formed through the hydrolysis process; subsequently, a micro-nanoscale organic or inorganic coating will be formed on the surface of the fabric through a condensation reaction [74]. This method has the advantages of a simple process, mild reaction conditions, high efficiency, and good film-forming properties. It plays an important role in the functionalization of textiles, such as wrinkle resistance, dyeing, UV protection, antistatic, antibacterial, flame retardant, and hydrophobic properties. The durable antibacterial and flame-retardant cotton fabrics were developed via simultaneous hydrolytic condensation of N_3_P_3_[NH(CH_2_)_3_Si(OC_2_H_5_)_3_]_6_ and polymerization of dopamine (PDA) on cotton fabric. Ag NPs were then introduced onto fabrics via in situ reactions with PDA. Considerable flame retardancy can be observed for treated cotton fabric even at a low loading (7.2%) of the hybrid coating. In addition, the antibacterial activity of the treated fabric reached 99.99% for both *S. aureus* and *E. coli*. The modification showed excellent durability and nearly intact antimicrobial properties, and flame retardancy was maintained after 30 washing cycles [75]. In another work, a multifunctional composite coating (APP @ SiO_2_-PDA @ Ag) composed of APP, PDA, PDMS-silica (PDMS-SiO_2_), and Ag NPs was constructed on the surface of PET fabrics. The APP @ SiO_2_-PDA @ Ag PET fabric showed an LOI of 29.0% and could self-extinguish in the VFT, and its PHRR and THR were 34% and 26% lower than those of the pure PET fabric. Notably, the multifunctional PET fabrics also exhibited excellent antibacterial activity against *E. coli* and *S. aureus* and superhydrophobicity (>150°). More importantly, the APP@SiO_2_-PDA@Ag-coated PET fabrics still maintained good flame retardant and antibacterial performances after multiple washing cycles [76] (Figure 7).

The introduction of functionalized trialkoxysilane as the sol-gel agent has made significant progress in the chemical modification process of textiles, which is beneficial for producing unique surface properties. A three-component equimolar sol mixture of SiF, 3-(trimethoxysilyl)-propyldimethyloctadecyl ammonium chloride (SiQ), P, and SiP was constructed on cotton fabric by the sol-gel method. The treated fabrics simultaneously achieved flame-retardant, antibacterial (bacterial reduction of 100%), and water-repellent properties due to the thermal stability of SiP, the antibacterial properties of SiQ, and the hydrophobicity of SiF [77]. Following this work, the same group further optimized the structure of the multifunctional coating to increase the washing speed of treated cotton fabrics. They applied the prepared Sto¨ber silica particles onto cotton fibers to form a particle-containing polysiloxane layer, which is based on tetraethyl orthosilicate, in the preparation work before the process of sol-gel. Eventually, the results showed enhanced washing fastness under the influence of the deposition of the silica particles. At the same time, it still exhibits excellent antibacterial activity, with R values of 81.6 and 100% for *E. coli* and *S. aureus* [14].

## 4. Conclusions and Perspectives

This review summarizes the different treatments and adaptable finishing agents to obtain flame-retardant, antibacterial, multi-functional fabrics. Through the latest decade of research, it was found that the treatment methods not only affect processing efficiency but also have strong ties with the final functional properties and wearability of fabric. At the same time, the relevant functional materials were further introduced here, including their source, characteristics, and mechanism. Moreover, the advantages and disadvantages of these treatment methods and finishing agents were briefly introduced, which can provide a basic reference for relevant research in this field.

Although finishing methods and agents have been developing rapidly, many shortcomings remain and need to be solved. (1) Improving durability. The functional-coating textile products inescapably undergo friction and water-washing during daily applications. It will lead to bad results from a damaged coating and decrease or even eliminate the effect of the functional fabrics. Therefore, it is proposed that more research focus be laid on the durability of the coating. (2) Developing environmentally friendly but high-performance finishing agents. It is an eternal task for the whole of humanity to promote sustainable development. However, the effect of most green functional treatments is greatly limited. Therefore, it is integral to spend effort researching innocuous technology and chemicals while developing high-performance fabrics. (3) Increasing yields and realizing industrialization earlier. Plenty of research just stays in the laboratory stage, and it can hardly be applied in practice. Increasing production could promote the industrialization process, and realizing industrialization earlier will improve the quality of life and accelerate social progress. (4) It is well known that dual-functional fabrics can be realized by a two-step or one-step method. The two-step method may suffer from functional antagonism when flame retardants and antimicrobial agents are utilized simultaneously, which is detrimental to the construction of bifunctional coatings. In addition, the one-step method is important in the design and synthesis of multifunctional compounds. Therefore, to achieve the multi-functionalization of textiles, the best efforts are needed to find the optimal process and functional materials.

The above challenges will be gradually overcome with the continuous development of science and technology as well as the appearance of innovative technologies and materials. Owing to their powerful functionality and portability, multifunctional fabrics have been increasing in popularity in markets and will have good development prospects for a long time in the future.

## Figures and Tables

**Table 1:** The flame retardancy and antibacterial effect of phenolic compounds with metal mordanting.

MPNs	Seif-Extinguish	Vertical Combustion Test	LOI (%)	*S. aureus* (%)	*E. coli* (%)	Reference
TA-Fe^2+^		B1	27.5	~98	~97	[48]
Tea Stem Extract-Fe^2+^	Yes	B1	~26.7	97	93	[49]
Tea Stem Extract-Fe^3+^	Yes	B1	~26.7	95	80	[49]
Tea Stem Extract-Ti^4+^	Yes	B1	~26.3	96	83	[49]
baicalin-Fe^2+^	No	B1	~27	~90	~93	[52]
baicalin-Ti^4+^	No	B1	~27	~91	~91	[52]
quercetin-Fe^2+^	No	B1	~27	~87	~90	[52]
quercetin-Ti^4+^	No	B1	~27.2	~86	~92	[52]
Rutin-Fe^2+^	No	B1	~26.8	~85	~90	[52]
Rutin-Ti^4+^	No	B1	~27	~87	~87.8	[52]
GSPs-Fe^2+^		B1	~27.8		99	[51]
GSPs-Fe^3+^		B1	~28		91	[51]
GSPs-Ti^4+^			~26.8		96	[51]

## Abbreviations

ASTM	American Society for Testing and Materials
GB/T	Guobiao Standards (Chinese National Standards)
IEC	International Electrotechnical Commission
ISO	International Organization for Standardization
NFPA	National (US) Fire Prevention Association
UL-94	Standard released by the Underwriters Laboratories (USA)

## References

[1] Li P., Wang B., Xu Y.-J., Jiang Z., Dong C., Liu Y., Zhu P. (2019). Ecofriendly Flame-Retardant Cotton Fabrics: Preparation, Flame Retardancy, Thermal Degradation Properties, and Mechanism. ACS Sustain. Chem. Eng..

[2] Costes L., Laoutid F., Brohez S., Dubois P. (2017). Bio-based flame retardants: When nature meets fire protection. Mater. Sci. Eng. R Rep..

[3] Qiu X., Li Z., Li X., Zhang Z. (2018). Flame retardant coatings prepared using layer by layer assembly: A review. Chem. Eng. J..

[4] Liang F., Xu Y., Chen S., Zhu Y., Huang Y., Fei B., Guo W. (2022). Fabrication of Highly Efficient Flame-Retardant and Fluorine-Free Superhydrophobic Cotton Fabric by Constructing Multielement-Containing POSS@ZIF-67@PDMS Micro-Nano Hierarchical Coatings. ACS Appl. Mater. Interfaces.

[5] Sykam K., Hussain S.S., Sivanandan S., Narayan R., Basak P. (2023). Non-halogenated UV-curable flame retardants for wood coating applications: Review. Prog. Org. Coat..

[6] Gao M., Wu W., Xu Z.-Q. (2013). Thermal degradation behaviors and flame retardancy of epoxy resins with novel silicon-containing flame retardant. J. Appl. Polym. Sci..

[7] Wang X., Hu Y., Song L., Xing W., Lu H. (2010). Thermal degradation behaviors of epoxy resin/POSS hybrids and phosphorus-silicon synergism of flame retardancy. J. Polym. Sci. Part B Polym. Phys..

[8] Lu Y., Zhao P., Chen Y., Huang T., Liu Y., Ding D., Zhang G. (2022). A bio-based macromolecular phosphorus-containing active cotton flame retardant synthesized from starch. Carbohydr. Polym..

[9] Song T., Li Z.S., Liu J.G., Yang S.Y. (2012). Novel phosphorus–silicon synergistic flame retardants: Synthesis and characterization. Chin. Chem. Lett..

[10] Zhang J., Chen B., Liu J., Zhu P., Liu Y., Jiang Z., Dong C., Lu Z. (2020). Multifunctional antimicrobial and flame retardant cotton fabrics modified with a novel N,N-di(ethyl phosphate) biguanide. Cellulose.

[11] Lin J., Chen X., Chen C., Hu J., Zhou C., Cai X., Wang W., Zheng C., Zhang P., Cheng J. (2018). Durably Antibacterial and Bacterially Antiadhesive Cotton Fabrics Coated by Cationic Fluorinated Polymers. ACS Appl. Mater. Interfaces.

[12] Wu M., Ma B., Pan T., Chen S., Sun J. (2016). Silver-Nanoparticle-Colored Cotton Fabrics with Tunable Colors and Durable Antibacterial and Self-Healing Superhydrophobic Properties. Adv. Funct. Mater..

[13] Magovac E., Vončina B., Budimir A., Jordanov I., Grunlan J.C., Bischof S. (2021). Environmentally Benign Phytic Acid-Based Nanocoating for Multifunctional Flame-Retardant/Antibacterial Cotton. Fibers.

[14] Vasiljević J., Zorko M., Štular D., Tomšič B., Jerman I., Orel B., Medved J., Kovač J., Simončič B. (2017). Structural optimisation of a multifunctional water- and oil-repellent, antibacterial, and flame-retardant sol–gel coating on cellulose fibres. Cellulose.

[15] Guo W., Wang X., Huang J., Zhou Y., Cai W., Wang J., Song L., Hu Y. (2020). Construction of durable flame-retardant and robust superhydrophobic coatings on cotton fabrics for water-oil separation application. Chem. Eng. J..

[16] Li N., Han H., Li M., Qiu W., Wang Q., Qi X., He Y., Wang X., Liu L., Yu J. (2021). Eco-friendly and intrinsic nanogels for durable flame retardant and antibacterial properties. Chem. Eng. J..

[17] Schartel B., Hull T.R. (2007). Development of fire-retarded materials—Interpretation of cone calorimeter data. Fire Mater..

[18] Li K., Mao S., Feng R. (2019). Estimation of Heat Release Rate and Fuel Type of Circular Pool Fires Using Inverse Modelling Based on Image Recognition Technique. Fire Technol..

[19] Laoutid F., Bonnaud L., Alexandre M., Lopez-Cuesta J.M., Dubois P. (2009). New prospects in flame retardant polymer materials: From fundamentals to nanocomposites. Mater. Sci. Eng. R Rep..

[20] Lin D., Zeng X., Li H., Lai X. (2018). Facile fabrication of superhydrophobic and flame-retardant coatings on cotton fabrics via layer-by-layer assembly. Cellulose.

[21] Reddy P.R.S., Agathian G., Kumar A. (2005). Ionizing radiation graft polymerized and modified flame retardant cotton fabric. Radiat. Phys. Chem..

[22] Liu Z., Xu M., Wang Q., Li B. (2017). A novel durable flame retardant cotton fabric produced by surface chemical grafting of phosphorus- and nitrogen-containing compounds. Cellulose.

[23] Jiang Z., Wang C., Fang S., Ji P., Wang H., Ji C. (2018). Durable flame-retardant and antidroplet finishing of polyester fabrics with flexible polysiloxane and phytic acid through layer-by-layer assembly and sol-gel process. J. Appl. Polym. Sci..

[24] Pan Y., Liu L., Zhang Y., Song L., Hu Y., Jiang S., Zhao H. (2019). Effect of genipin crosslinked layer-by-layer self-assembled coating on the thermal stability, flammability and wash durability of cotton fabric. Carbohydr. Polym..

[25] Bentis A., Boukhriss A., Grancaric A.M., El Bouchti M., El Achaby M., Gmouh S. (2019). Flammability and combustion behavior of cotton fabrics treated by the sol gel method using ionic liquids combined with different anions. Cellulose.

[26] Lin D., Zeng X., Li H., Lai X., Wu T. (2019). One-pot fabrication of superhydrophobic and flame-retardant coatings on cotton fabrics via sol-gel reaction. J. Colloid Interface Sci..

[27] Ling C., Guo L. (2021). An eco-friendly and durable multifunctional cotton fabric incorporating ZnO and a branched polymer. Cellulose.

[28] Yu R., Tian M., Qu L., Zhu S., Ran J., Liu R. (2018). Fast and simple fabrication of SiO_2_/poly(vinylidene fluoride) coated cotton fabrics with asymmetric wettability via a facile spray-coating route. Text. Res. J..

[29] Attia N.F., Morsy M.S. (2016). Facile synthesis of novel nanocomposite as antibacterial and flame retardant material for textile fabrics. Mater. Chem. Phys..

[30] Zheng C., Sun Y., Cui Y., Yang W., Lu Z., Shen S., Xia Y., Xiong Z. (2021). Superhydrophobic and flame-retardant alginate fabrics prepared through a one-step dip-coating surface-treatment. Cellulose.

[31] Wei D., Dong C., Liu J., Zhang Z., Lu Z. (2019). A Novel Cyclic Polysiloxane Linked by Guanidyl Groups Used as Flame Retardant and Antimicrobial Agent on Cotton Fabrics. Fibers Polym..

[32] Moazami A., Montazer M. (2015). A novel multifunctional cotton fabric using ZrO2NPs/urea/CTAB/MA/SHP: Introducing flame retardant, photoactive and antibacterial properties. J. Text. Inst..

[33] Makhlouf G., Abdelkhalik A., Ameen H. (2022). Preparation of highly efficient chitosan-based flame retardant coatings with good antibacterial properties for cotton fabrics. Prog. Org. Coat..

[34] Yang T.-T., Guan J.-P., Tang R.-C., Chen G. (2018). Condensed tannin from Dioscorea cirrhosa tuber as an eco-friendly and durable flame retardant for silk textile. Ind. Crops Prod..

[35] Okeil A.A. (2008). Citric Acid Crosslinking of Cellulose Using TiO_2_Catalyst by Pad-Dry-Cure Method. Polym.-Plast. Technol. Eng..

[36] Ahmed M.T., Morshed M.N., Farjana S., An S.K. (2020). Fabrication of new multifunctional cotton–modal–recycled aramid blended protective textiles through deposition of a 3D-polymer coating: High fire retardant, water repellent and antibacterial properties. New J. Chem..

[37] Singh N., Sheikh J. (2021). Sustainable development of mosquito-repellent, flame-retardant, antibacterial, fragrant and antioxidant linen using microcapsules containing Thymus vulgaris oil in in-situ generated chitosan-phosphate. Cellulose.

[38] El-Shafei A., ElShemy M., Abou-Okeil A. (2015). Eco-friendly finishing agent for cotton fabrics to improve flame retardant and antibacterial properties. Carbohydr. Polym..

[39] Dhineshbabu N.R., Manivasakan P., Karthik A., Rajendran V. (2014). Hydrophobicity, flame retardancy and antibacterial properties of cotton fabrics functionalised with MgO/methyl silicate nanocomposites. RSC Adv..

[40] Vasiljević J., Tomšič B., Jerman I., Orel B., Jakša G., Kovač J., Simončič B. (2014). Multifunctional superhydrophobic/oleophobic and flame-retardant cellulose fibres with improved ice-releasing properties and passive antibacterial activity prepared via the sol–gel method. J. Sol-Gel Sci. Technol..

[41] Alongi J., Carosio F., Malucelli G. (2014). Current emerging techniques to impart flame retardancy to fabrics: An overview. Polym. Degrad. Stab..

[42] Alongi J., Carosio F., Frache A., Malucelli G. (2013). Layer by Layer coatings assembled through dipping, vertical or horizontal spray for cotton flame retardancy. Carbohydr. Polym..

[43] Cheng X.-W., Tang R.-C., Guan J.-P., Zhou S.-Q. (2020). An eco-friendly and effective flame retardant coating for cotton fabric based on phytic acid doped silica sol approach. Prog. Org. Coat..

[44] Li P., Wang B., Liu Y.Y., Xu Y.J., Jiang Z.M., Dong C.H., Zhang L., Liu Y., Zhu P. (2020). Fully bio-based coating from chitosan and phytate for fire-safety and antibacterial cotton fabrics. Carbohydr. Polym..

[45] Li P., Liu C., Wang B., Tao Y., Xu Y.-J., Liu Y., Zhu P. (2021). Eco-friendly coating based on an intumescent flame-retardant system for viscose fabrics with multi-function properties: Flame retardancy, smoke suppression, and antibacterial properties. Prog. Org. Coat..

[46] Hou F., Zhu M., Liu Y., Zhu K., Xu J., Jiang Z., Wang C., Wang H. (2022). A self-assembled bio-based coating with phytic acid and DL-arginine used for a flame-retardant and antibacterial cellulose fabric. Prog. Org. Coat..

[47] Liu Y., Zhang J., Ren Y., Zhang G., Liu X., Qu H. (2022). Biomaterial arginine encountering with UV grafting technology to prepare flame retardant coating for polyacrylonitrile fabric. Prog. Org. Coat..

[48] Song J., Feng H., Wu M., Chen L., Xia W., Zhang W. (2020). Preparation and characterization of arginine-modified chitosan/hydroxypropyl methylcellose antibacterial film. Int. J. Biol. Macromol..

[49] Tang H., Zhang P., Kieft T.L., Ryan S.J., Baker S.M., Wiesmann W.P., Rogelj S. (2010). Antibacterial action of a novel functionalized chitosan-arginine against Gram-negative bacteria. Acta Biomater..

[50] Su Z., Han Q., Zhang F., Meng X., Liu B. (2020). Preparation, characterization and antibacterial properties of 6-deoxy-6-arginine modified chitosan. Carbohydr. Polym..

[51] Jiang Z., Hu Y., Zhu K., Li Y., Wang C., Zhang S., Wang J. (2021). Self-assembled bio-based coatings for flame-retardant and antibacterial polyester–cotton fabrics. Text. Res. J..

[52] Lv Z., Hu Y.-T., Guan J.-P., Tang R.-C., Chen G.-Q. (2019). Preparation of a flame retardant, antibacterial, and colored silk fabric with chitosan and vitamin B2 sodium phosphate by electrostatic layer by layer assembly. Mater. Lett..

[53] Dong A., Wang Y.J., Gao Y., Gao T., Gao G. (2017). Chemical Insights into Antibacterial N-Halamines. Chem. Rev..

[54] Ren H., Du Y., Su Y., Guo Y., Zhu Z., Dong A. (2018). A Review on Recent Achievements and Current Challenges in Antibacterial Electrospun N-halamines. Colloid Interface Sci. Commun..

[55] Li S., Lin X., Liu Y., Li R., Ren X., Huang T.-S. (2019). Phosphorus-nitrogen-silicon-based assembly multilayer coating for the preparation of flame retardant and antimicrobial cotton fabric. Cellulose.

[56] Xiao M., Guo Y., Zhang J., Liu Y., Ren Y., Liu X. (2021). Diethylene triamine penta methylene phosphonic acid encountered silver ions: A convenient method for preparation of flame retardant and antibacterial lyocell fabric. Cellulose.

[57] Xu D., Wang S., Hu J., Liu Y., Jiang Z., Zhu P. (2021). Enhancing antibacterial and flame-retardant performance of cotton fabric with an iminodiacetic acid-containing N-halamine. Cellulose.

[58] Keskin D., Zu G., Forson A.M., Tromp L., Sjollema J., van Rijn P. (2021). Nanogels: A novel approach in antimicrobial delivery systems and antimicrobial coatings. Bioact. Mater..

[59] Kaur I., Bhati P., Sharma B. (2014). Antibacterial, flame retardant, and physico-chemical properties of cotton fabric graft copolymerized with a binary mixture of acrylonitrile and 4-vinylpyridine. J. Appl. Polym. Sci..

[60] Dong C., He P., Lu Z., Wang S., Sui S., Liu J., Zhang L., Zhu P. (2017). Preparation and properties of cotton fabrics treated with a novel antimicrobial and flame retardant containing triazine and phosphorus components. J. Therm. Anal. Calorim..

[61] Jiang T., Liu L., Yao J. (2011). In situ deposition of silver nanoparticles on the cotton fabrics. Fibers Polym..

[62] Nozari B., Montazer M., Mahmoudi Rad M. (2021). Stable ZnO/SiO_2_ nano coating on polyester for anti-bacterial, self-cleaning and flame retardant applications. Mater. Chem. Phys..

[63] Kale R.D., Soni M., Potdar T. (2019). A flame retardant, antimicrobial and UV protective polyester fabric by solvent crazing route. J. Polym. Res..

[64] Abu Elella M.H., Goda E.S., Yoon K.R., Hong S.E., Morsy M.S., Sadak R.A., Gamal H. (2021). Novel vapor polymerization for integrating flame retardant textile with multifunctional properties. Compos. Commun..

[65] Cai H., Liu Z., Xu M., Chen L., Chen X., Cheng L., Li Z., Dai F. (2021). High performance flexible silk fabric electrodes with antibacterial, flame retardant and UV resistance for supercapacitors and sensors. Electrochim. Acta.

[66] Elsayed E.M., Attia N.F., Alshehri L.A. (2020). Innovative Flame Retardant and Antibacterial Fabrics Coating Based on Inorganic Nanotubes. Chem. Sel..

[67] Nabipour H., Wang X., Rahman M.Z., Song L., Hu Y. (2020). An environmentally friendly approach to fabricating flame retardant, antibacterial and antifungal cotton fabrics via self-assembly of guanazole-metal complex. J. Clean. Prod..

[68] Yu Z., Liu J., He H., Ma S., Yao J. (2021). Flame-retardant PNIPAAm/sodium alginate/polyvinyl alcohol hydrogels used for fire-fighting application: Preparation and characteristic evaluations. Carbohydr. Polym..

[69] Zhang W., Yang Z.-Y., Tang R.-C., Guan J.-P., Qiao Y.-F. (2020). Application of tannic acid and ferrous ion complex as eco-friendly flame retardant and antibacterial agents for silk. J. Clean. Prod..

[70] Cheng T.-H., Liu Z.-J., Yang J.-Y., Huang Y.-Z., Tang R.-C., Qiao Y.-F. (2019). Extraction of Functional Dyes from Tea Stem Waste in Alkaline Medium and Their Application for Simultaneous Coloration and Flame Retardant and Bioactive Functionalization of Silk. ACS Sustain. Chem. Eng..

[71] Jiang P., Zhu Y., Wu Y., Lin Q., Yu Y., Yu W., Huang Y. (2021). Synthesis of flame-retardant, bactericidal, and color-adjusting wood fibers with metal phenolic networks. Ind. Crops Prod..

[72] Guo L., Yang Z.-Y., Tang R.-C., Yuan H.-B. (2020). Grape Seed Proanthocyanidins: Novel Coloring, Flame-Retardant, and Antibacterial Agents for Silk. ACS Sustain. Chem. Eng..

[73] Zhou Y., Tang R.-C., Xing T., Guan J.-P., Shen Z.-H., Zhai A.-D. (2019). Flavonoids-metal salts combination: A facile and efficient route for enhancing the flame retardancy of silk. Ind. Crops Prod..

[74] Malucelli G. (2016). Surface-Engineered Fire Protective Coatings for Fabrics through Sol-Gel and Layer-by-Layer Methods: An Overview. Coatings.

[75] Li Y., Wang B., Sui X., Xie R., Xu H., Zhang L., Zhong Y., Mao Z. (2018). Durable flame retardant and antibacterial finishing on cotton fabrics with cyclotriphosphazene/polydopamine/silver nanoparticles hybrid coatings. Appl. Surf. Sci..

[76] Li Q., Zhang S., Mahmood K., Jin Y., Huang C., Huang Z., Zhang S., Ming W. (2021). Fabrication of multifunctional PET fabrics with flame retardant, antibacterial and superhydrophobic properties. Prog. Org. Coat..

[77] Vasiljević J., Tomšič B., Jerman I., Orel B., Jakša G., Simončič B. (2014). Novel multifunctional water- and oil-repellent, antibacterial, and flame-retardant cellulose fibres created by the sol–gel process. Cellulose.

